# Transient receptor potential ion channels and cerebral stroke

**DOI:** 10.1002/brb3.2843

**Published:** 2022-12-16

**Authors:** Qin'yi Xu, Yan Zou, Zeng'li Miao, Lei Jiang, Xu'dong Zhao

**Affiliations:** ^1^ Department of Neurosurgery The Affiliated Wuxi No. 2 Hospital of Nanjing Medical University Wuxi China; ^2^ Department of Neurosurgery The Affiliated Jinling Hospital Medical School of Nanjing University Nanjing China

**Keywords:** cerebral stroke, glia, neuron, transient receptor potential ion channels

## Abstract

**Methods:**

The databases Pubmed, and the National Library of Medicine were searched for literature. All papers on celebral stroke and transient receptor potential ion channels were considered.

**Results:**

Stroke is the second leading cause of death and disability, with an increasing incidence in developing countries. About 75 per cent of strokes are caused by occlusion of cerebral arteries, and substantial advances have been made in elucidating mechanisms how stroke affects the brain. Transient receptor potential (TRP) ion channels are calcium‐permeable channels highly expressed in brain that drives Ca^2+^ entry into multiple cellular compartments. TRPC1/3/4/6, TRPV1/2/4, and TRPM2/4/7 channels have been implicated in stroke pathophysiology.

**Conclusions:**

Although the precise mechanism of transient receptor potential ion channels in cerebral stroke is still unclear, it has the potential to be a therapeutic target for patients with stroke if developed appropriately. Hence, more research is needed to prove its efficacy in this context.

## Graphical abstract

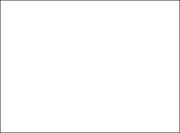

## 1 INTRODUCTION

Stroke is one of the leading causes of long‐term disability and the fifth leading cause of death among adults in the United States (Writing Group et al., 2016). Cerebral stroke is known to occur when blood supply to a particular brain region is disrupted, and it consists of two different types, ischemic and hemorrhagic. Ischemic stroke accounts for 75% of cases and is caused by the obstruction of a cerebral blood vessel, either by plaque formation in situ or by embolus formation elsewhere, which subsequently travels to the brain. Hemorrhagic stroke, the second type, accounts for 25% of cases and is caused by the rupture of a cerebral blood vessel.

Over the past decade, there have been rapid advances in stroke research. Several mechanisms have been implicated, alone or in conjunction, in stroke‐induced neuronal cell death, including excitotoxicity, free radicals release, protein misfolding, mitochondrial death pathways, apoptosis, necrosis, autophagy, and inflammation (Gleichman & Carmichael, 2014; Rubinsztein et al., 2015; Sabri et al., 2013; Szydlowska & Tymianski, 2010). Besides neuronal loss, damage to or loss of astrocytes can also contribute to cerebral injury, and intracellular accumulation of Ca^2+^ plays a toxic role in this procedure (Chen et al., 2014; Patel et al., 2013; Song & Yu, 2014). Recently, TRP ion channels have been shown to regulate Ca^2+^ homeostasis and participate in stroke pathophysiology.

The mammalian TRP channel superfamily encompasses 28 identified Ca^2+^‐permeable channels with diverse cellular distributions and physiological functions (Nilius & Owsianik, 2011; Song & Yuan, 2010). TRP channels are categorized into two comprehensive groups known as group 1 and group 2, as well as an additional family known as TRPY (Table 1). These channels are divided into seven subfamilies based on their structural homology and function: TRPC (canonical), TRPV (vanilloid), TRPM (melastatin), TRPA (ankyrin like), TRPML (mucolipin), TRPP (polycystin), and TRPN (*Drosophila* NOMPC). All TRP channels contain six‐transmembrane segments and a pore‐forming loop between the fifth and sixth segments (Nilius & Owsianik, 2011). TRP channels, except TRPM4 and TRPM5, are nonselective Ca^2+^‐permeable cation channels that play a critical role in numerous cellular processes via the modulation of cytosolic free [Ca^2+^]*_i_* by acting as Ca^2+^‐permeable channels in the plasma membrane or via modulating the driving force for Ca^2+^ influx by altering the membrane potential (Nilius et al., 2007). For each group of TRP ion channels described below, TRP channels involved in stroke have been highlighted, and their potential as novel therapeutic targets to combat different pathogenesis of stroke was examined, with a particular focus on ischemia.

**TABLE 1:** Transient receptor potential channels groups and subfamilys

TRP channels superfamily					
Group 1		Group 2		Group TRPY	
TRPA	TRPC	TRPML	TRPP	TRPY	
TRPM	TRPN				
TRPS	TRPV				
TRP VL					

## 2 TRPC CHANNELS

TRPC channels include seven members that associate as homo‐ or heterotetramers (Putney, 2005; Schaefer, 2005). Within the seven TRPC members (TRPC1‐7), TRPC1 forms heteromers with TRPC4 or TRPC5, while TRPC3, TRPC6, and TRPC7 form heteromers exhibiting distinct properties from homomultimers (Goel et al., 2002; Strubing et al., 2001). TRPC1, TRPC3, and TRPC4 are involved in store‐operated Ca^2+^ entry (SOCE), of which TRPC1 is the most well‐established channel contributing to SOCE. TRPC channels may be activated directly by diacylglycerol or indirectly via calcium release from the endoplasmic reticulum following stimulation of the inositol triphosphate receptor (Kress et al., 2008; Sours‐Brothers et al., 2009). Only those TRPC channels with proven roles in cerebral stroke are discussed below.

### 2.1 TRPC1

TRPC1 reportedly plays a protective role against ischemia/reperfusion (I/R)‐induced neurological injury by suppressing reactive oxygen species (ROS) generation (Xu et al., 2018). TRPC1 expression was downregulated in both in vivo and in vitro I/R models. TRPC1 knockdown exacerbated I/R‐induced brain infarction, edema, memory impairment, neurological deficits, and oxidative stress in vivo. TRPC1 downregulation resulted in the activation of Nox4‐containing Nicotinamide Adenine Dinucleotide Phosphate (NADPH) oxidase, which interacts with Nox4 to facilitate Nox4 degradation. In addition, it was shown to potentiate the translocation of p47phox and p67phox and the interaction between Nox4, p47phox, and p67phox (Xu et al., 2018).

### 2.2 TRPC3

TRPC3 has been implicated in I/R pathogenesis. Brain injury was ameliorated in TRPC3/6/7 knockout (KO) mice subjected to I/R. The phosphorylation of nuclear factor‐κB (NF‐kB) and expression of pro‐apoptotic protein Bcl‐2‐associated X (Bax) were reduced in brain tissues of I/R‐injured TRPC3/6/7 KO mice, accompanied by increased phosphorylation of anti‐apoptotic protein Bcl‐2 and protein kinase B (AKT). Astrocytes isolated from TPRC3/6/7 KO mice subjected to I/R exhibited enhanced Bcl‐2 and p‐AKT expression, reduced Bax expression, and NF‐kB phosphorylation (Chen et al., 2017).

Notably, TRPC3 is implicated in astrogliosis after intracerebral hemorrhage (ICH). Thrombin, the major blood‐derived serine protease, leaks out of blood vessels into the brain parenchyma following disruption of the blood–brain barrier (BBB) and aggravates brain injury by accelerating astrogliosis after stroke (Nishino et al., 1993). In cultured astrocytes derived from rats and humans, thrombin treatment enhanced TPRC3 expression through protease‐activated receptor 1, extracellular signal‐regulated protein kinase (ERK), c‐JunNH2‐terminal kinase, and NF‐κB signaling (Rutowski & Peterson, 1993; Shirakawa et al., 2010). Upregulated TRPC3 protein leads to increased Ca^2+^ influx in astrocytes and regulates gliosis (Shibasaki et al., 2010). In vivo experiments, blocking TRPC3 with a specific antagonist, that is, ethyl‐1‐(4‐(2,3,3‐trichloroacrylamide)phenyl)−5‐(trifluoromethyl)−1H‐pyrazole‐4‐carboxylate (Pyr3), improved functional outcomes and attenuated astrogliosis (Munakata et al., 2013). Therefore, TRPC3 may act as a new therapeutic target for both ischemic and hemorrhagic brain injury.

### 2.3 TRPC4

TRPC4 positively contributes to ischemic brain damage. Infarct areas were markedly smaller in the brain of TRPC4^−/−^ mice than those in wild‐type (WT) mice, especially with a shorter period of ischemia. In particular, 30 min of ischemia followed by 24 h of reperfusion resulted in scarcely detectable infarct areas in TRPC4^−/−^ brains, while they were readily visible in WT specimens; however, the protective effect of TRPC4 was attenuated after prolonged ischemia (120 min). In vitro, neurons from WT mice were more vulnerable to the 2 h bicarbonate buffer exposure than those derived from TRPC4^−/−^ mice, irrespective of oxygenation status (Jeon et al., 2020). Followed by reoxygenation and nutrient replenishment for 24 h, TRPC4 KO neurons exhibited an obvious refeeding‐dependent increase in cell death only when oxygen and glucose deprivation (OGD) treatment was applied. These data suggest that targeting the TRPC4 channel might be beneficial in ischemia therapy but additional studies are needed to establish the appropriate intervention strategies.

### 2.4 TRPC6

TRPC6 upregulation or downregulation can activate or suppress Ca^2+^ overload induced by NMDA (Li et al., 2012). The precise mechanisms include the phosphorylation of NMDA receptors by increasing ERK, calcineurin, and Ca^2+^/calmodulin‐dependent protein kinase (CaMK) activities (Jia et al., 2007; Liu & Ji, 2012; Tai et al., 2008). Abnormal TRPC6 function is detrimental to neurons after stroke, given that Ca^2+^ overload contributes to cell death following ischemia. TRPC6 protein levels were reduced in a transient middle cerebral artery occlusion (tMCAO) model; however, mRNA levels remained were unaltered after reperfusion owing to calpain‐mediated proteolysis (Du et al., 2010; Lin et al., 2013, 2013). In addition, TRPC6 protein expression was significantly downregulated during I/R injury in cultured astrocytes. Calpains are Ca^2+^‐dependent non‐lysosomal cysteine proteases. Under ischemic conditions, calpain activation is initiated by excessive Ca^2+^ influx through NMDA receptors in neurons (Goll et al., 2003). Therefore, TRPC6 degradation after stroke is calcium dependent. Appropriate TRPC6 protein levels for post‐stroke neuroprotection are also maintained by the cyclic AMP response‐element binding protein (CREB) signaling pathway (Du et al., 2010). Inhibiting TPRC6 degradation may promote p‐CREB, thereby upregulating the expression of downstream neuroprotective molecules, such as brain‐derived neurotrophic factor (BDNF) and Bcl‐2 (Kitagawa, 2007).

A growing number of studies have focused on stem cell therapy for ischemic stroke. In vivo research has indicated that bone marrow‐derived stromal cells (BMSCs) can improve stroke outcomes (Yang et al., 2014). TRPC6 channel has been implicated in neuroprotection toward BMSCs and during oxiracetam combination therapies in acute brain IR damage (Wang et al., 2019). In addition, TRPC6 overexpression in BMSCs improved neuronal functions in rats after ischemia via the BDNF/CREB pathway (Li et al., 2019). In addition, the inflammatory response participates in the pathophysiology of ischemic stroke. Interleukin (IL)‐17A, a pro‐inflammatory cytokine, is associated with brain IR injury (Gelderblom et al., 2012). Another study has reported that TRPC6 may act downstream of IL‐17A, suggesting that IL‐17A can promote TRPC6 degradation, thus exacerbating cerebral IR injury (Zhang et al., 2014). TRPC3/6/7 KO reportedly decreased pro‐apoptotic factors and NF‐κB expression, decreased NF‐κB nuclear translocation, and upregulated activation of , ultimately inducing resistance against cerebral IR injury (Chen et al., 2017).

Accumulated evidence has confirmed the beneficial role of TRPC6 post‐stroke using hyperforin, resveratrol, peanuts, (‐)‐epigallocatechin‐3‐gallate, calycosin, monoterpene oxide 1,8‐cineole (cineole), and tetramethylpyrazine (Guo et al., 2017; Lin et al., 2013, 2013; Meng et al., 2021; Shao et al., 2017). In MCAO rats, resveratrol or hyperforin and cineole enhanced TPRC6 expression and p‐CREB. Therefore, the infarct volume decreased, accompanied by enhanced neurological function. The protective role of TPRC6 was also demonstrated following the application of HYP9 (a selective TRPC6 agonist). HYP9 dose‐dependently inhibited TPRC6 downregulation and reduced apoptosis, cytotoxicity and inflammatory responses in astrocytes by reducing NF‐kB nuclear translocation and phosphorylation in I/R injury, whereas SKF96365 (SKF, a TRPC antagonist) aggravated this damage in vitro (Liu et al., 2020). These findings suggest that TRPC6 may act as a potential therapeutic target, and additional studies are warranted to comprehensively elucidate the underlying intervention mechanism.

## 3 TRPV CHANNELS

TRPV channels are composed of six members, and TRPV1, TRPV2, and TRPV4 are predominantly distributed in the central nervous system (CNS). TRPV1 is a prototype receptor activated by heat, and synthetic, or endogenous vanilloids (Caterina et al., 1997). TRPV2 shares approximately 50% sequence identity with TRPV1 and exert distinct cellular functions from TPRV1 (Cohen et al., 2015). Recent reports on the implications of TRPV1, TRPV2, and TRPV4 in stroke are described below.

### 3.1 TRPV1

Several recent studies have indicated that TRPV1 participates in stroke pathophysiology, but the precise mechanism remains elusive. Following induction of transient ischemia using the MCA occlusion model, TRPV1‐KO mice had lower infarct volumes and, neurological and motor deficits than WT mice. A TRPV1 antagonist, capsazepine (20 nmol), injected intracerebroventricularly 30 min before the onset of ischemia, attenuated neurological and motor deficits and decreased infarct size instead of influencing cerebral blood flow in the occluded MCA territory. Capsazepine failed to afford neuroprotection against ischemic brain damage in TRPV1‐KO mice. Therefore, the TRPV1 channel is upregulated after ischemic stroke and causes larger infarctions and neurological and motor deficits after brain ischemia (Miyanohara et al., 2015). The effect of TRPV1 in ischemia has been confirmed in the permanent MCAO model. TRPV1 antagonism (AMG9810) significantly attenuated the infarct volume when compared with that in the control group. In addition, AMG9810 decreased neurological deficits 7 days after cerebral ischemia, as well as improved scores in the ledged beam‐walking test (Hakimizadeh et al., 2017).

In addition, an in vitro OGD/reoxygenation (OGD/R) model has been established to induce cerebral I/R injury. In the in vitro OGD/R microglial model, TRPV1 could partly activate OGD/R‐induced microglial autophagy, which impedes oxidative stress‐induced injury and apoptosis (Huang et al., 2021; Lin et al., 2022). Based on the previous result, 6‐gingerol treatment, which possesses anti‐apoptotic and anti‐inflammatory effects, was employed and was shown to significantly attenuate cerebral infarct volume, improve brain edema and neurological scores, and reverse brain histomorphological damage after I/R injury (Luo et al., 2021).

In a rodent tMCAO model, capsaicin‐mediated activation of TRPV1 immediately after reperfusion reportedly induced reversible hypothermia and afforded neuroprotection before the infarction fully developed (Cao et al., 2014). The observed neuroprotection could be attributed to hypothermia rather than central or peripheral effects of TRPV1 activation (Muzzi et al., 2012). In addition to acute stroke management, long‐term TRPV1 activation may help reduce stroke risk. Chronic dietary capsaicin significantly relaxed basilar arteries, reduced intracranial arterial hypertrophy, and induced endothelial nitric oxide synthase (eNOS) phosphorylation in stroke‐prone spontaneously hypertensive rats (Xu et al., 2011). In neonatal rats, pretreatment with capsaicin afforded neuroprotection against hypoxia/ischemia‐induced brain damage in neonatal rats Khatibi et al. (2011). Thus, the potential effects of TRPV1 for stroke therapy remain controversial and warrant careful investigation.

### 3.2 TRPV2

Astrocytes have been reported to regulate CNS homeostasis and influence the prognosis of ischemic injury. Regulation of Ca^2+^ signaling in astrocytes is a promising strategy for stroke, and TRPV2 is an important osmotic balance regulator, mainly expressed in cortical astrocytes. Moreover, OGD/R enhanced TRPV2 expression and increased intracellular Ca^2+^ levels. SiRNA‐mediated blockade of TRPV2 promoted astrocyte proliferation and increased nerve growth factor (NGF) mRNA expression via c‐JunN‐terminalkinase (JNK) phosphorylation. Thus, targeting TRPV2 channels in astrocytes may be a potential new therapeutic strategy for ischemic stroke (Zhang et al., 2016).

### 3.3 TRPV4

TRPV4 is also involved in the pathophysiology of ischemia. In a global hypoxia/ischemia rat model, TRPV4 expression rapidly decreased in neurons and gradually increased in astrocytes, suggesting that TRPV4 may participate in neuronal death during the acute phase of stroke and in astrogliosis during the chronic phase (Butenko et al., 2012). Compared with WT mice, TRPV4^−/−^ mice exhibited reduced ischemia‐induced lesion volume and decreased water content and Evans blue leakage in the ipsilateral hemisphere, accompanied by milder neurological symptoms (Tanaka et al., 2020). HC‐067047, a specific TRPV4 antagonist, reduced brain infarction following 60 min of MCAO (Jie et al., 2016). GSK1016790A (a TRPV4 agonist) dose‐dependently induced hippocampal neuronal death, along with increased phosphorylation of the NR2B subunit of the *N*‐methyl‐d‐aspartate receptor (NMDAR) (Jie et al., 2016). TRPV4 is also activated by arachidonic acid, cell swelling, or epoxyeicosatrienoic acids in neurons, which are associated with cerebral ischemia (Seubert et al.., 2007). Activation of the TRPV4 channel upregulated Ca^2+^ entry via NMDARs and required the NR2B subunit (Li et al., 2013). TRPV4 channels contribute to extracellular glutamate accumulation during peri‐infarct depolarizations (Rakers et al., 2017). Furthermore, TRPV4 activation can depolarize the resting membrane potential, facilitating presynaptic glutamate release (Shibasaki et al., 2007). As Ca^2+^ entry via NMDARs is the major pathway leading to neuronal death following stroke, TRPV4 channel activation could aggravate glutamate excitotoxicity. In addition to affecting NMDARs, Ca^2+^ entry via TRPV4 also stimulated the production of ROS and nitric oxide (NO), downregulated p‐Akt, and upregulated p‐ERK, thus exacerbating neuronal injury post‐stroke (Bubolz et al., 2012; Donko et al., 2010; Jie et al., 2016). GSK1016790A and HC‐067047 dose‐dependently induced or inhibited apoptosis in the mouse hippocampi (GSK‐injected mice). The phosphorylated p38 mitogen‐activated protein kinase (p‐p38 MAPK) and cleaved caspase‐3 protein levels were markedly increased or decreased, while the Bcl‐2/Bax protein ratio was decreased or increased.

In astrocytes, TRPV4 expression or activity was gradually enhanced post‐stroke. In hypoxia‐treated astrocytes, spontaneous [Ca^2+^]_i_ increased, indicating Ca^2+^ entry via TRPV4 channels. As Ca^2+^ levels play a critical role in several cellular functions, TRPV4 activation could affect the Ca^2+^‐dependent release of neurotransmitters, growth factors, and cytokines from reactive astrocytes following ischemia.

TRPV4 also plays a crucial role in vascular remodeling. The TRPV4 agonist 4α‐phorbol 12,13‐didecanoate (4α‐PDD) reduced infarct volume and improved functional outcomes in rats subjected to transient brain ischemia. Activation of the TRPV4 channel significantly increased eNOS expression and phosphorylation (serine 1177) in the ischemic region. Expression levels of vascular endothelial growth factor A (VEGFA) and VEGF receptor‐2 were significantly elevated in the 4α‐PDD‐treated animals, particularly exhibiting an increase in the proangiogenic VEGFA164a isoform and a decrease in the antiangiogenic VEGFA165b isoform. 4α‐PDD treatment increased microvessel density (Chen et al., 2018). Parenchymal microvessels in the ischemic lesion were compressed and narrowed by the swollen endfeet of astrocytes in WT mice, but these effects markedly disappeared in TRPV4^−/−^ mice (Tanaka et al., 2020).

Brain edema is an important pathological process that occurs during stroke. Matrix metalloproteinases (MMPs) are upregulated after TRPV4 activation in lung tissues and digest the endothelial basal lamina to destroy the BBB, causing vasogenic brain edema. At 48 h post‐MCAO, TRPV4 antagonist HC‐067047 decreased the brain water content and Evans blue extravasation. In addition, HC‐067047 attenuated the increased MMP‐2/9 protein expression and only increased MMP‐9 activity in the hippocampus of MCAO mice. HC‐067047 also attenuated downregulated zonula occludens‐1 (ZO‐1) and occludin protein in MCAO mice. Moreover, the TRPV4 agonist GSK1016790A exerted the reverse effect. In an in vitro model of ischemic stroke‐induced edema, OGD‐induced swelling of brain slices was attenuated by pharmacologically blocking or genetically knocking out TRPV4 (Hoshi et al., 2018).

ICH is a severe subtype of stroke with high morbidity and mortality rates. Administration of GSK1016790A, a selective TRPV4 agonist, attenuated neurological and motor deficits in collagenase‐induced ICH. Inhibition was completely reversed in TRPV4‐KO mice. GSK1016790A significantly upregulated the expression of c‐Fos, a marker of neuronal activity (Asao et al., 2020).

Therefore, blocking TRPV4 might be neuroprotective post‐stroke but its clinical effect necessitates further investigation.

## 4 TRPM CHANNELS

The TRPM subfamily comprises eight members, consisting of four six‐transmembrane domain subunits, resulting in homomeric or heteromeric channels. Several TRPM channels, such as TRPM2, TRPM4, and TRPM7 have been implicated in stroke.

### 4.1 TRPM2

TRPM2 is activated by intracellular Adenosine Diphosphate Ribose (ADPR), hydrogen peroxide, heat, and ROS (Nilius et al., 2007; Wu et al., 2010). In a tMCAO model, TRPM2 mRNA was upregulated during the chronic period from 1 to 4 week post‐surgery (Fonfria et al., 2006). TRPM2 is expressed in hippocampal, cortical, and microglial cells; however, the effect of TRPM2 on ischemia remains controversial (Bai & Lipski, 2010; Kaneko et al., 2006; Olah et al., 2009). In neurons, TRPM2 is responsible for H_2_O_2_‐induced Ca^2+^ entry and subsequent neuronal death (Kaneko et al., 2006). Furthermore, activation of the TRPM2 channel increased Ca^2+^ influx, mitochondrial membrane depolarization‐induced free oxygen radicals, release of apoptotic factors (including caspases 3 and 9), and eventual cell death in ischemia‐induced hippocampal neuronal injury (Akpinar et al., 2016). Recently, numerous antioxidants, like edaravone and *N*‐acetylcysteine (NAC), were found to protect nerve cells against cerebral ischemia injury by blocking TRPM2‐mediated Ca^2+^ influx and increasing neuronal survival (Pun et al., 2009; Sun et al., 2018). Another study reported that H_2_O_2_ increased synaptic excitability in TRPM2^−/−^ CA1 neurons when compared with WT neurons. The increased excitability resulted from changes in the GluN2A/GluN2B ratio. These changes affect downstream Akt and ERK pathways. A decrease in postsynaptic density‐95 kDa (PSD‐95) and an increase in p‐GSK3β were observed in the TRPM2^−/−^ hippocampus (Alim et al., 2013). Ischemia‐induced TRPM2 activation increased extracellular zinc ions expression. In addition, TRPM2 channel KO attenuated increased Zn^2+^, lysosomal dysfunction, and neuronal cell death (Ye et al., 2014). TRPM2 knockdown also reduced OGD‐induced neuronal injury, which could be attributed to reduced oxidative stress levels, downregulated mitochondrial membrane potentials, decreased intracellular calcium concentrations, attenuated NLRP3 inflammasome activation, and inhibition of apoptosis (Pan et al., 2020).

In microglia, upregulation of TRPM2 channels continually occurs weeks after focal cerebral ischemia (Fonfria et al., 2006). TRPM2 plays a prominent role in generating nitric oxide (NO) in microglial cells. Following exposure to ischemia, microglial cells undergo multiple pathophysiological changes from resting cell to fully activated, phagocytizing tissue macrophages. Bacterial lipopolysaccharide (LPS) and interferon‐γ (IFN‐γ)‐mediated microglial activation and induced TRPM2‐mediated Ca^2+^ signaling, thus causing Pyk2 activation and upregulation of downstream MAPK and JNK signaling, thereby upregulating inducible nitric oxide synthase (iNOS) and CXCL‐2 mRNA in microglia (Miyake et al., 2014). LPS‐induced TRPM2 KO astrocytes showed decreased levels of inflammation mediators (interleukin [IL])‐1β, IL‐6, and TNF‐α level) (Zhu et al., 2019). In addition, TRPM2‐activated peripheral immune cells contribute to ischemic brain injury. In in vivo experiments using bone marrow chimeric mice, TRPM2 directly resulted in neutrophil migration and, to a lesser extent, macrophage migration into the ischemic hemispheres. Furthermore, TRPM2 can aggravate brain damage in these cell types. TRPM2 knockdown reduced TNF‐α secretion in neutrophils, macrophages, and dendritic cells after ischemia (Gelderblom et al., 2014). Therefore, TRPM2, as a potential target for stroke therapy, needs to establish a balance between effects on microglia and neurons. Indeed, pretreatment with clotrimazole, a nonselective TRPM2 blocker, reduced cell death following OGD, as well as the glutamate‐mediated excitotoxic effect on cultured cerebellar granule cells (Isaev et al., 2002). However, blockers of TRPM2 channels, clotrimazole, *N*‐(p‐amylcinnomoyl) anthranilic acid, or flufenamic acid, failed to protect pyramidal neurons from exogenous H_2_O_2_‐induced cell death.

Notably, no difference was observed in infarct volume between TRPM2 KO and WT mice following permanent ischemia. Accordingly, the role of the TRPM2 channel during I/R indicates that TRPM2 KO can only protect against transient brain damage (followed by reperfusion) but not permanent damage (no reperfusion) (Nakayama et al., 2013). The potential therapeutic role of TRPM2 blockade in stroke remains controversial.

### 4.2 TRPM4

TRPM4, a monovalent nonselective cation channel, is proposed to form the SUR1/TRPM4 channel complex as a pore, along with SUR1 (Kurland et al., 2013). In the CNS, SUR1 is only expressed in specific neurons instead of astrocytes or capillaries. In animal models and human stroke tissues, SUR1 upregulation was observed in neurons, astrocytes, and endothelial tissues (Mehta et al., 2013; Simard et al., 2006, 2010). Following ischemia, the TRPM4 channel colocalized and associated with Sur1 within ischemic endothelial cells and neurons. SUR1/TRPM4 channel opening leads to unchecked Na^+^ influx, which causes oncotic cell death (Simard et al., 2012). The SUR1/TRPM4 channel also contributes to vasogenic edema by upregulating perivascular TNF, extravasation of serum immunoglobulin G, and associated inflammation (Mehta et al., 2015). Therefore, blocking SUR1 with sulfonylureas or glibenclamide could improve stroke outcomes (Simard et al., 2014). Stroke also upregulates TRPM4 expression in the vascular endothelium (Loh et al., 2014; Mehta et al., 2015). Inhibition of TRPM4 using siRNAs or glibenclamide facilitated angiogenesis while capillary fragmentation disappeared, resulting in reduced infarction and enhanced motor functions (Gerzanich et al., 2009; Mehta et al., 2015).

Endothelial upregulation of TRPM4 occurs as early as 2 h post‐stroke induction. TRPM4 successfully reduced edema and salvaged the functionally active brain. In a permanent ischemia model, both edema and significant reductions in metabolic activity were detected in large areas of the brain tissue, which was not observed in transient models with or without TRPM4 knockdown, indicating the potential survival of tissues salvaged by TRPM4 inhibition during stroke reperfusion (Chen et al., 2019).

M4P, a TRPM4‐specific antibody binding to the channel pore region, may decrease the TRPM4 current and cellular expression, thereby preventing hypoxia‐induced cell swelling. In the I/R rat model, M4P application attenuated reperfusion injury by maintaining BBB integrity and facilitating functional recovery (Chen et al., 2019).

### 4.3 TRPM7

TRPM7 is a ubiquitously expressed nonselective cation channel that possesses protein kinase activity (Wu et al., 2010). The role of TRPM7 in stroke has been extensively studied. TRPM7 activity is greatly enhanced after ischemia/hypoxia, possibly due to both TRPM7 protein upregulation and channel potentiation owing to the stroke‐induced acidic environment (Aarts et al., 2003; Jiang et al., 2008). The excessive influx of permeable metal ions such as Ca^2+^, Mg^2+^, and Zn^2+^, through TRPM7 can be highly toxic to cells (Inoue et al., 2010; Lee et al., 2002). In addition to metal ion overloading, TRPM7 can induce neuronal ROS generation post‐hypoxia (Aarts et al., 2003). NGF regulates both TRPM7 function and expression via the PI3K signaling pathway (Jiang et al., 2008; Tian et al., 2007). TRPM7 is an MLKL downstream target that mediates Ca^2+^ entry and causes TNF‐associated necroptosis (Cai et al., 2014). In addition, TRPM7 suppression attenuated neuronal death and improved ischemia‐induced deficits in long‐term potentiation and fear‐associated or spatial–navigational memory (Sun et al., 2009).

Carvacrol can reportedly inhibit TRPM7 currents in vitro and reduce OGD‐induced neuronal injury. TRPM7 protein levels in the ipsilateral hemisphere significantly increased in the contralateral hemisphere 24 h post‐hypoxic‐ischemic injury. Pretreatment with carvacrol reduced brain infarct volume 24 h post‐hypoxic‐ischemic injury and improved neurobehavioral outcomes. Furthermore, carvacrol pretreatment in vivo resulted in fewer TUNEL‐positive cells in the brain, decreased cleaved caspase‐3, and increased the Bcl‐2/Bax protein ratio and p‐Akt/t‐Akt ratio (Chen et al., 2015).

## 5 CONCLUSION

Over the past few years, there has been steady progress in clarifying the pathophysiology of stroke. In addition, the contribution of TRP channels in stroke pathophysiology has been increasingly documented. The TRPC1/3/4/6, TRPV1/2/4, and TRPM2/4/7 channels have been extensively investigated focusing on their effects on neurons and gliocytes post‐stroke. However, studies on the role of TRP channels in neuronal death post‐stroke are lacking. Therefore, comprehensively understanding the function of these channels in stroke will not only enrich our knowledge of stroke pathophysiology, but may also provide novel therapeutic targets. However, the side effects of targeting the TRP channels in stroke therapy should be further examined. Future studies attempting to consolidate the adverse effects will allow a more comprehensive awareness of the roles of TRP channels in stroke, ultimately resulting in the development of novel therapeutic strategies.

## CONFLICT OF INTEREST

The authors declare that they have no competing interests.

### PEER REVIEW

The peer review history for this article is available at https://publons.com/publon/10.1002/brb3.2843.

## Data Availability

Data available on request from the authors
